# Retraction: Different Amyloid-β Self-Assemblies Have Distinct Effects on Intracellular Tau Aggregation

**DOI:** 10.3389/fnmol.2021.764608

**Published:** 2021-09-23

**Authors:** Frontiers Editorial Office

The journal retracts the 08 November 2019 article cited above.

Following publication, concerns were raised regarding the integrity of the images in the published figures. The authors failed to provide a satisfactory explanation during the investigation, which was conducted in accordance with Frontiers' policies.

This retraction was approved by the Chief Editors of Frontiers in Molecular Neuroscience and the Chief Executive Editor of Frontiers. The authors did not agree to this retraction.

